# microRNA-181a-5p promotes fibroblast differentiation of mesenchymal stem cells in rats with pelvic floor dysfunction

**DOI:** 10.1016/j.clinsp.2024.100428

**Published:** 2024-07-06

**Authors:** YongHong Zhang, HaiYang Yu, JianChao Li

**Affiliations:** a Department of Pediatrics, Muping District Hospital of Traditional Chinese Medicine, Yantai City, Shandong Province, China; b Department of Gynecology, Muping District Hospital of Traditional Chinese Medicine, Yantai City, Shandong Province, China

**Keywords:** Pelvic floor dysfunction, miR-181a-5p, MFN1, Bone marrow-derived mesenchymal stem cells

## Abstract

The use of stem cells capable of multilineage differentiation in treating Pelvic Floor Dysfunction (PFD) holds great promise since they are susceptible to entering connective tissue of various cell types and repairing damaged tissues. This research investigated the effect of microRNA-181a-5p (miR-181a-5p) on Bone Marrow Mesenchymal Stem Cells (BMSCs) in rats with PFD. BMSCs were transfected and analyzed for their fibroblast differentiation ability. miR-181a-5p, MFN1, and fibroblast-related genes were quantitatively analyzed. Whether MFN1 is a target gene of miR-181a-5p was predicted and confirmed. The efficacy of BMSCs *in vivo* rats with PFD was evaluated by measuring Leak Point Pressure (LPP), Conscious Cystometry (CMG), hematoxylin and eosin staining, and Masson staining. The present results discovered that miR-181a-5p was up-regulated and MFN1 was down-regulated during the differentiation of BMSCs into fibroblasts. Fibroblast differentiation of BMSCs was promoted after miR-181a-5p was induced or MFN1 was suppressed, but it was suppressed after miR-181a-5p was silenced. miR-181a-5p improved LPP and conscious CMG outcomes in PDF rats by targeting MFN1 expression, thereby accelerating fibroblast differentiation of BMSCs. In brief, miR-181a-5p induces fibroblast differentiation of BMSCs in PDF rats by MFN1, potentially targeting PDF therapeutics.

## Highlights

• miR-181a-5p promotes fibroblast differentiation of BMSCs.
• miR-181a-5p promotes fibroblast differentiation of BMSCs by targeting MFN1.
• BMSCs containing miR-181a-5p improve PFD in SD rats by targeting MFN1 expression, thereby accelerating the fibroblast differentiation of BMSCs.

## Introduction

Pelvic Floor Dysfunction (PFD) is a disorder of abnormal anatomy or function of pelvic organs due to weakening of the supporting tissues of the pelvic floor and dislocation of pelvic organs.^1^ PFD can lead to various diseases, such as pelvic organ prolapse, chronic pain syndromes, urinary and fecal incontinence, defecation dysfunction, or lower urinary tract sensory.^2^^,^^3^ Symptomatic and conservative treatment is the mainstay of care for patients with PFD; surgery is considered only for those who fail or refuse conservative treatment, but up to 20‒30% of patients with recurrence will require re-surgery.^4^ Although artificial biomesh during surgery can improve long-term recovery, and the mesh can also cause pain, erosion, and scarring in about 30 percent of cases.^5^ Therefore, alternative approaches are required to enhance tissue repair and regeneration in PFD.

The use of stem cells capable of multilineage differentiation in treating PFD holds great promise since they are susceptible to entering connective tissue of various cell types and repairing damaged tissues.^6^^,^^7^ Bone Marrow Mesenchymal Stem Cells (BMSCs) are easy to be isolated and cultured, have strong differentiation ability, and secrete biological active factors beneficial to tissue repair.^8^^,^^9^ Current studies have identified that transplantation of BMSCs can alleviate PFD in an experimental model.^10^^,^^11^ For this reason, in-depth research on how BMSCs repair PFD is required.

MicroRNAs (miRNAs) guide post-transcriptional repression by pairing with the mRNAs of protein-coding genes, further regulating protein production and cellular biological functions.^12^ miRNAs regulate key pathways involved in stem cell function^13^ and interestingly, BMSCs modified by miRNAs have recently demonstrated excellent therapeutic results in PDF.^14^ miR-181a-5p is an important miRNA involved in orthopedic diseases, including femoral head necrosis,^15^ osteoarthritis,^16^ and osteoporosis,^17^ and miR-181a-5p could regulate BMSC apoptosis and differentiation.^17^

It is speculated that miR-181a-5p alleviates PDF by inducing differentiation in BMSCs. This research was designed based on a rat model of PFD to evaluate the efficacy of BMSCs containing miR-181a-5p, aiming to provide a clinical theoretical basis and guidance for stem cell therapy of PFD.

## Methods

### Isolation and culture of BMSCs

All animal procedures followed the recommendations of the National Institutes of Health's Guide for the Care and Use of Laboratory Animals and ARRIVE guidelines. This study protocol was approved by the Muping District Hospital of Traditional Chinese Medicine (n° MP20190621). Femurs of 6-month-old female Sprague-Dawley rats (200‒250g) were made into a cell suspension with 5‒10 mL of ice-cold Iscove's Modified Dulbecco's Medium (IMDM), centrifuged at 150 × g for 5 min, and resuspended in IMDM. The cell suspension was placed in Percol Separation Solution (1.073 g/mL, Sigma-Aldrich), centrifuged at 400 × g, and maintained in IMDM containing 20% FBS and 1% streptomycin/penicillin (Sigma-Aldrich). With the medium renewed after 24h, cells were grown to 80% confluence and sub-cultured. Adherent fibroblast-like cells after 2 passages were BMSCs.^18^

### BMSCs identification

BMSCs were trypsinized and resuspended in 4% FBS-PBS. FITC-labeled anti-human CD44, CD90, CD73, and CD45 antibodies (eBioscience, CA, USA) were utilized to identify BMSCs. Data acquisition and analysis were conducted by flow cytometry using FACSDiva (Canto, BD Biosciences, CA, USA) and FlowJo software (Tree Star, OR, USA), respectively.^14^

### MTT assay

BMSCs at passage 3 (2 × 10^4^ cells/well) were cultured for 7d and supplemented with MTT solution (20 μL, 5 mg/mL, Sigma-Aldrich) for 4h. Then, the dissolved sample collected by 150 μL of dimethyl sulfoxide was conditioned to absorbance analysis at 490 nm on a microplate (Thermo Fisher Scientific).

### Adipogenic and osteogenic differentiation of BMSCs

BMSCs at passage 3 with 80% confluence were cultured in an adipogenic differentiation solution (High Glucose [HG]-DMEM containing 10% FBS, 10 μg/mL insulin [Sigma-Aldrich], 0.5 mM isobutylmethylxanthine, 1 Μm dexamethasone, and 200 μM indomethacin) for 3d and in an adipogenic differentiation solution (HG-DMEM plus 10% FBS and 10 μg/mL insulin) for 1d. The above procedure was repeated in a total of 3 cycles, and BMSCs were maintained in an adipogenic differentiation solution for 2d.

The osteogenic induction medium was prepared with 10% FBS, 1 μM dexamethasone, 10 mM sodium β-phosphate, and 50 mg/L vitamin C (Sigma-Aldrich) and refreshed every 72h. Adipogenic differentiation and osteogenic differentiation required 14 days each. The resulting cells were viewed after Oil red O staining and Alizarin red staining,^19^ respectively.

### Fibroblast differentiation of BMSCs

BMSCs at passage 3 (3 × 10^4^ cells/mL) were cultured in HG-DMEM containing 10% FBS, TGF-β1 at 15 ng/mL, bFGF at 20 ng/mL, and dexamethasone at 0.1 μmoL/L). The medium was renewed every 24h. The fibroblast differentiation process took 14 d.

### RNA interference

BMSCs (8 × 10^4^ cells/well) were RNA-modified using Lipofectamine 2000 (Invitrogen). The interference plasmids included miR-181a-5p mimic, inhibitor, and control (miR-NC), as well as short hairpin RNA (shRNA) and control (sh-RNA) targeting MFN1 (GenePharma, Shanghai, China). miRNA transfection concentration was maintained at 20 nmoL/L. MFN1 shRNA (Sequence: CCGGGCTCCCATTGATTCCAATACTCGAGTATTGGAATCATAATGGGAGC TTTTTG).

### RT-qPCR

Total RNA from BMSCs was extracted with Trizol reagent (Invitrogen) and conditioned to reverse transcription of mRNA and miRNA using PrimeScript RT kit (Takara, Tokyo, Japan) and miRNA First Strand Synthesis kit (Takara, Japan), respectively. With the SYBR Green kit (Thermo Fisher Scientific) and the Mx3005P QPCR system (Agilent Technologies, CA, USA), PCR was implemented. β-actin and U6 were regarded as internal references.^20^ The primer sequences are shown in Table 1.

**Table 1:** Primers.

Genes	Primers (5′–3′)
U6	Forward: 5′-CTCGCTTCGGCAGCACA-3′
	Reverse: 5′-AACGCTTCACGAATTTGCGT-3′
miR-181a-5p	Forward: 5′-CGGCAACATTCAACGCTGT-3′
	Reverse: 5′-GTGCAGGGTCCGAGGTATTC-3′
MFN1	Forward: 5′-AGCGGGATTGGTCACACAAC-3′
	Reverse: 5′-CCTTCGGTCATAAGGTAGGCTT-3′
α‐SMA	Forward: 5′-AACTAAAGGAGCTGCTGACCC-3′
	Reverse: 5′-TGTTGCTGTCCAAGTTGCTC-3′
Collagen I	Forward: 5′-ATCAGCCCAAACCCCAAGGAGA-3′
	Reverse: 5′-CGCAGGAAGGTCAGCTGGATAG-3′
β-actin	Forward: 5′-AGGGAAATCGTGCGTGACAT-3′
	Reverse: 5′-GAACCGCTCATTGCCGATAG-3′

Note: miR-181a-5p, microRNA-181a-5p; MFN1, Mitofusin 1; α‐SMA, α-Smooth Muscle Actin.

### Immunoblot analysis

Total protein was extracted with 500 μL RIPA lysis buffer (Beyotime), loaded on 8% SDS-PAGE gels (Solarbio), transferred to PVDF membranes (Invitrogen), and blocked with 5% skim milk. Rabbit antibodies against α-SMA, collagen I, and MFN1 (1:1000, Abcam) were combined with the membranes, which were then mixed with goat-rabbit secondary antibody (1:10000) for 2 hours and detected by ECL kit (34080, Thermo Fisher Scientific). For data quantification, ImageJ software was utilized.^21^

### Binding relation analysis

TargetScan software was utilized for target gene analysis. The 3′-UTR region of MFN1 containing the target or mutated sequence of miR-181a-5p was cloned into pGL4 (Promega, WI, USA) to construct MFN1-wt-3′-UTR and MFN1-mut-3′-UTR. In BMSCs, miR-181a-5p mimics or mimic NC, in combination with the generated plasmids was transfected, followed by data analysis by a dual luciferase reporter assay (Promega).

### Rat PFD model

After 14 days after vaginal dilation, PFD was induced by injection with normal saline into the weakest area of the pelvis (female SD rats, 6-months old, *n* = 12 group). A solution (500 μL) containing 8 × 10^5^ BMSCs not treated or pre-transfected with mimic-NC or miR-181a-5p mimic was injected through the tail vein into PFD rats. Normal rats without any intervention served as a control.^22^

For PFD induction, an 18F catheter was inserted into the rat vagina and then secured with a 3‒0 silk single suture. A Foley balloon was attached to a pressure transducer (0.15 kg) to compress the pelvic floor for 4h.

After 14d of vaginal dilation, the success of PFD modeling was checked by conscious Cystometry (CMG) and Leak Point Pressure (LPP) tests, which were also suitable for assessing the efficacy of BMSCs in PFD rats after 7d of various treatments.^18^

### CMG test

PFD rats were inserted with a bladder catheter (PE-50) which was connected to a syringe pump (KD Scientific, PA, USA) and pressure transducer (Grass Instruments, RI, USA). Normal saline was injected at a rate of 5 mL/h. Urinary contractions were measured by force transducers (Grass instrument). The mean bladder baseline pressure, peak bladder pressure, void volume, and urethral pressure increase were recorded.^14^

### LPP test

PFD rats were inserted with a bladder catheter which was connected to a pressure transducer and flow pump. After intraperitoneal injection of urethane (1.2 g/kg), the bladder was squeezed until emptied, pumped with normal saline at 5 mL/h to 0.3 mL, and pressed until the bladder and urethra leaked out of the saline. At the first leakage, abdominal pressure was removed to record the peak pressure. LPP=peakbladderpressure−baselinebladderpressure.^14^

### Histopathology

After the tests, the completely resected urethra, vagina, fascia, and bladder tissue were fixed with 4% paraformaldehyde overnight and made into 5 μm sections for HE and Masson staining^19^ and were observed under a light microscope.

### Statistical analysis

SPSS 21.0 was feasible for data analysis. The Kolmogorov-Smirnov test showed that the data were normally distributed, and the results were expressed as mean ± standard deviation. One-way ANOVA, followed by LSD-t was utilized to compare multiple groups. Enumeration data shown as rate or percentage were assessed by the Chi-Square test; p was a two-sided test, and *p* < 0.05 was considered statistically significant.

## Results

### BMSC identification

**Fig. 1:**
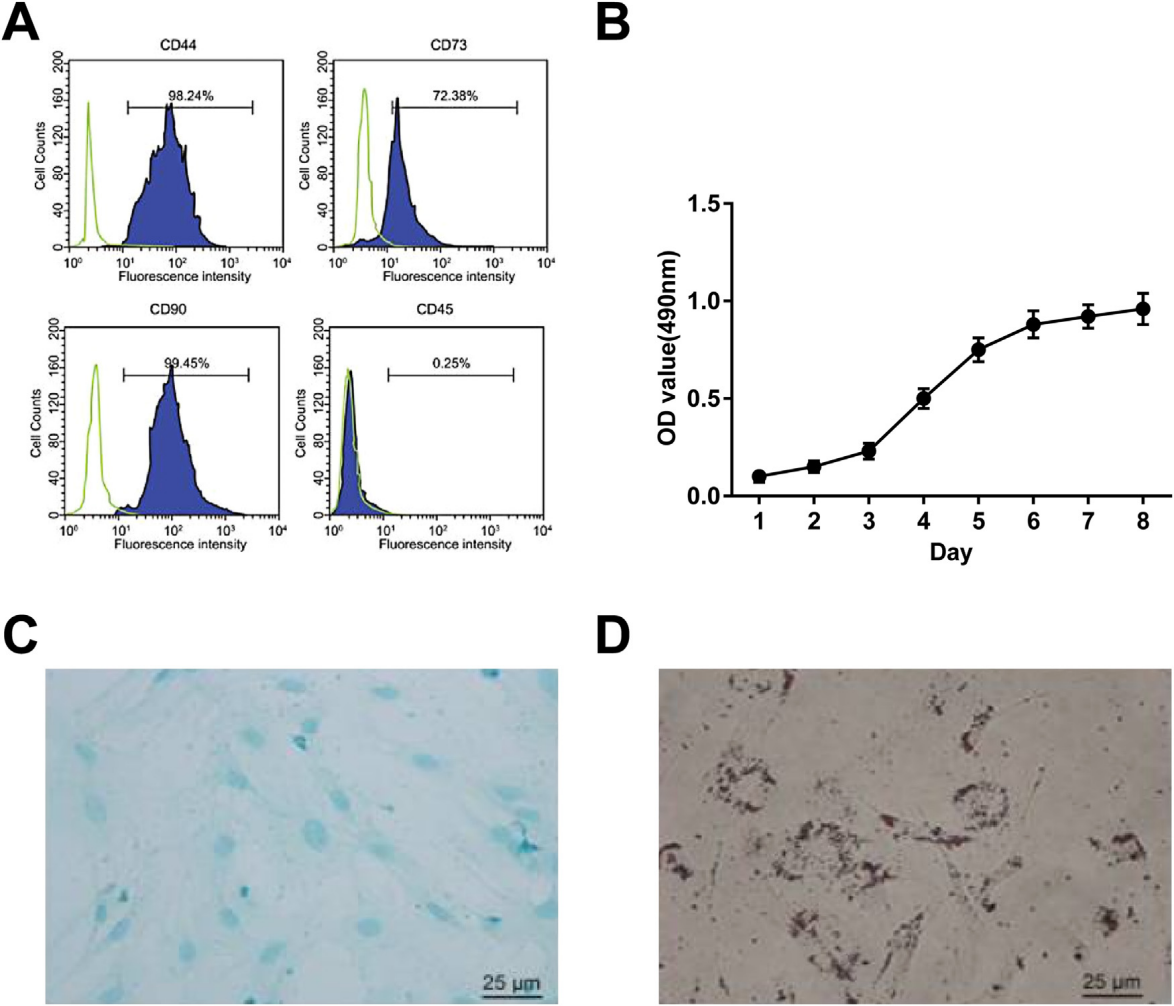
Isolation and identification of BMSCs. (A) CD44, CD73, CD90, and CD45 detected by flow cytometry; (B) Growth curve of BMSCs; (C) Oil red O of BMSCs after adipogenic induction (400 ×); (D) Alizarin red staining of BMSCs after osteogenic differentiation (400 ×); * *p <* 0.05, *n =* 3.

BMSCs were isolated by the whole bone marrow adhesion method and identified by flow cytometry. CD44, CD73, and CD90 (mesenchymal cell markers) positive staining and CD45 (hematopoietic cell marker) negative staining were seen (Fig. 1 A). MTT assay showed that BMSCs were grown in an S-curve, from the incubation period (the first 2d) and logarithmic growth phase (3^rd^ day) to the peak (7^th^ day) (Fig. 1 B). After induction, the isolated BMSCs had bidirectional adipogenic and osteogenic differentiation (Fig. 1 C, D).

### miR-181a-5p and MFN1 levels in BMSCs

**Fig. 2:**
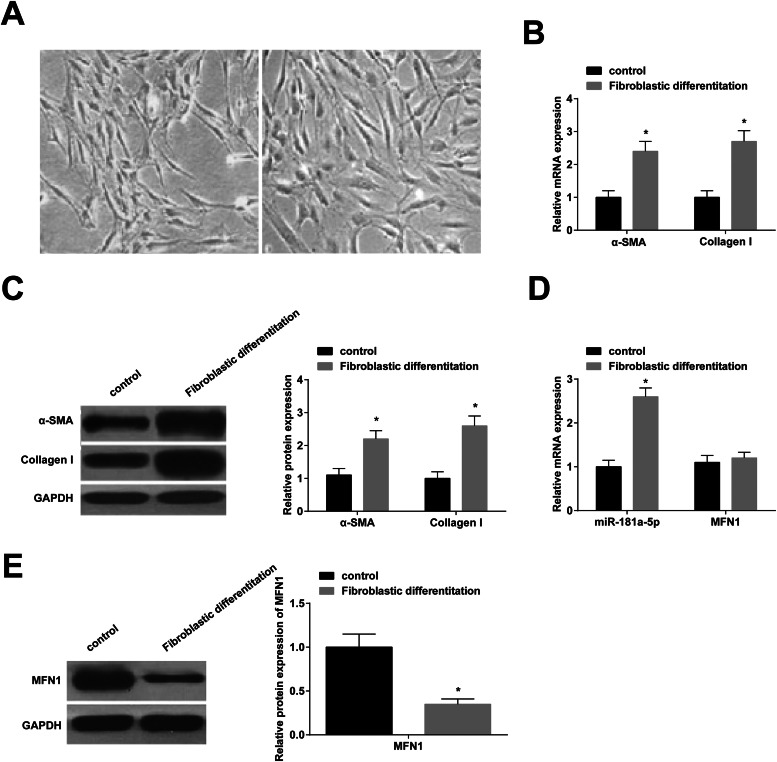
miR-181a-5p and MFN1 levels in BMSCs. (A) Morphology of BMSCs before and two weeks after fibroblast induction; (B/C) α‐SMA and Collagen I expression levels; (D) Quantitative miR-181a-5p and MFN1 mRNA expression; (E) Quantitative MFN1 protein expression; A‒F magnification: 100 ×; scale: 20 μm; * *p <* 0.05, *n =* 3.

At day 14 of fibroblast differentiation, BMSCs became more plump, spindle-shaped, and possessed more cytoplasmic (Fig. 2 A). RT-qPCR showed that fiber-related genes (α-SMA and Collagen I) were higher after induction (Fig. 2 B, C), which indicated that BMSCs could differentiate into fibroblasts. Interestingly, after fibroblast induction, miR-181a-5p expression was up-regulated (Fig. 2 D), and MFN1 protein expression was down-regulated (Fig. 2 E), while MFN1 mRNA expression was not significantly different (Fig. 2 D).

### miR-181a-5p promotes fibroblast differentiation of BMSCs

**Fig. 3:**
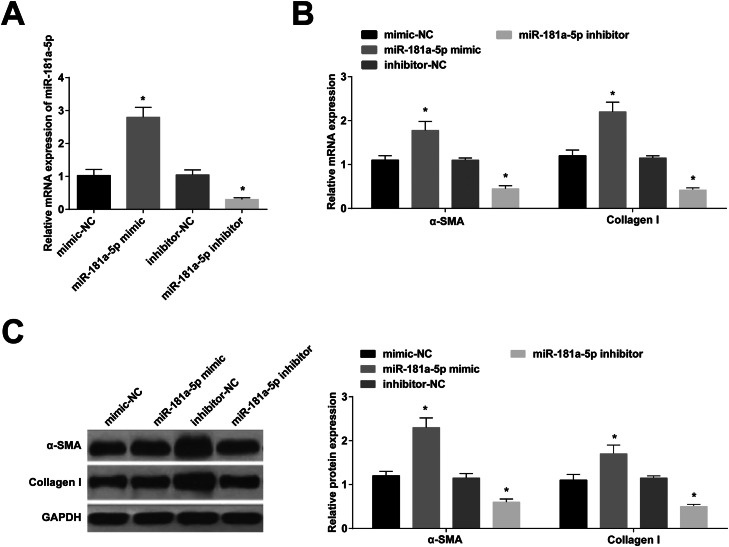
miR-181a-5p mimic promotes fibroblast differentiation of BMSCs. (A) miR-181a-5p expression in BMSCs. (B/C) α-SMA and Collagen I in BMSCs after fibroblast differentiation; * *p <* 0.05, *n =* 3.

miR-181a-5p mimic was modified in BMSCs, leading to increased miR-181a-5p expression, while the opposite was true after miR-181a-5p inhibitor modification (Fig. 3 A). Expression of α-SMA and collagen I was increased by miR-181a-5p up-regulation; expression of α-SMA and collagen I was suppressed by miR-181a-5p down-regulation (Fig. 3 B, C).

### miR-181a-5p promotes fibroblast differentiation of BMSCs by targeting MFN1

**Fig. 4:**
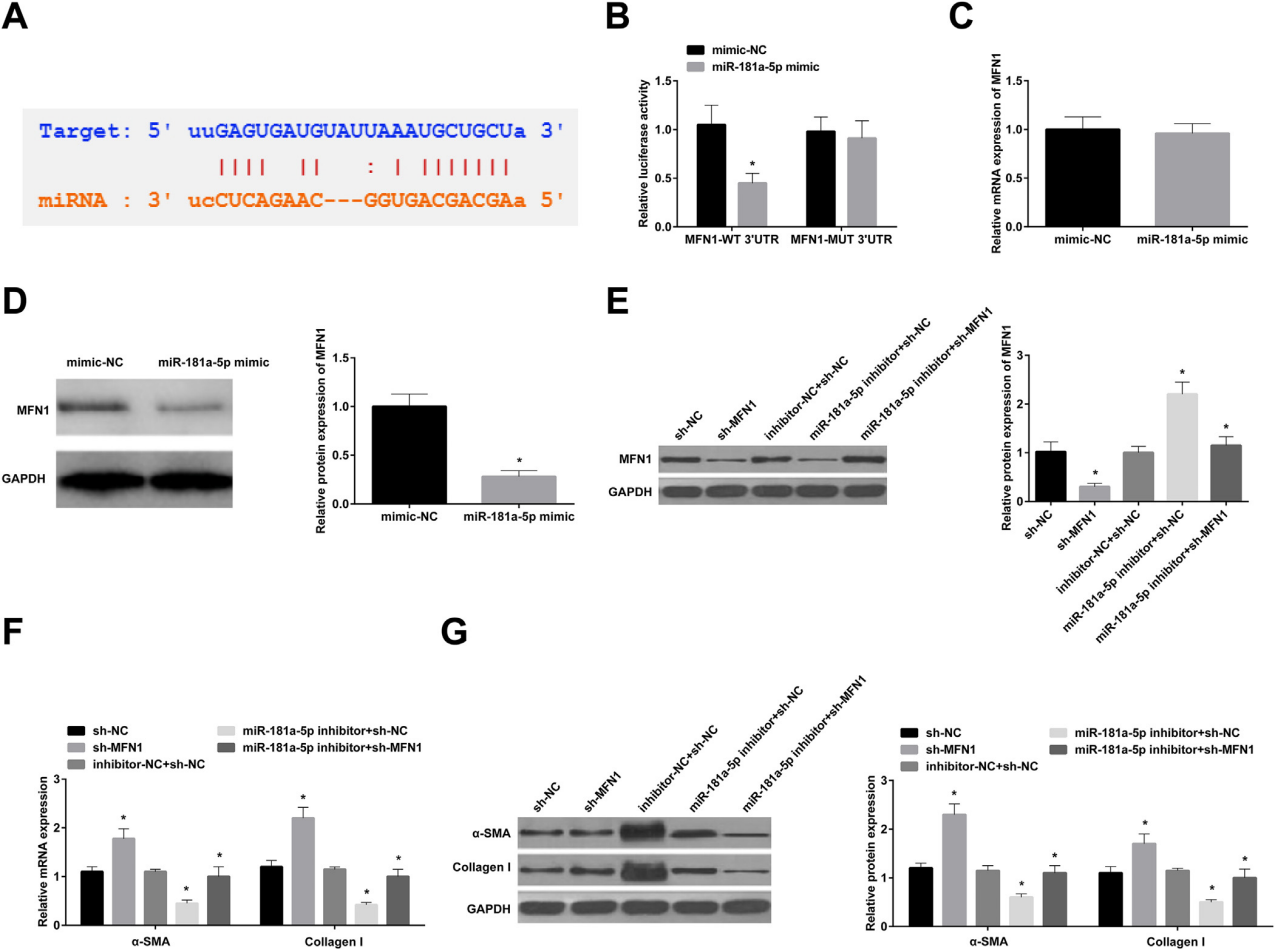
miR-181a-5p promotes fibroblast differentiation of BMSCs by targeting MFN1. (A) Bioinformatics website predicted the binding site between MFN1 and miR-181a-5p; (B) Dual-luciferase reporter gene assay to evaluate the interaction between MFN1 and miR-181a-5p; (C/D) MFN1 expression in BMSCs stably transfected with miR-181a-5p mimic; (E) MFN1 protein expression in BMSCs; (F/G) α-SMA and Collagen I expression in BMSCs after fibroblast differentiation; * *p <* 0.05, *n =* 3.

Bioinformatics analysis software revealed multiple complementary binding sites between miR-181a-5p and the 3′UTR of MFN1 (Fig. 4 A). Dual-luciferase reporter gene results are shown in Fig. 4 B. miR-181a-5p effectively reduced the luciferase activity of MFN1-WT-3′UTR but not that of MFN1-MUT 3′UTR. Next, in BMSCs stably transfected with miR-181a-5p mimic, MFN1 mRNA expression was not altered, but its protein expression was inhibited (Fig. 4 C, D). MFN1 protein expression was lowered in BMSCs transfected with sh-MFN1; and miR-181a-5p inhibitor-mediated increased protein expression of MFN1 was repressed by sh-MFN1 (Fig. 4 E). In addition, MFN1 knockdown alone increased α-SMA and Collagen I levels in BMSCs, while miR-181a-5p inhibitor-mediated changes in the two proteins were partially mitigated by MFN1 knockdown (Fig. 4 F, G).

### BMSCs containing miR-181a-5p improve PFD in rats by targeting MFN1 expression and accelerating the fibroblast differentiation of BMSCs

**Fig. 5:**
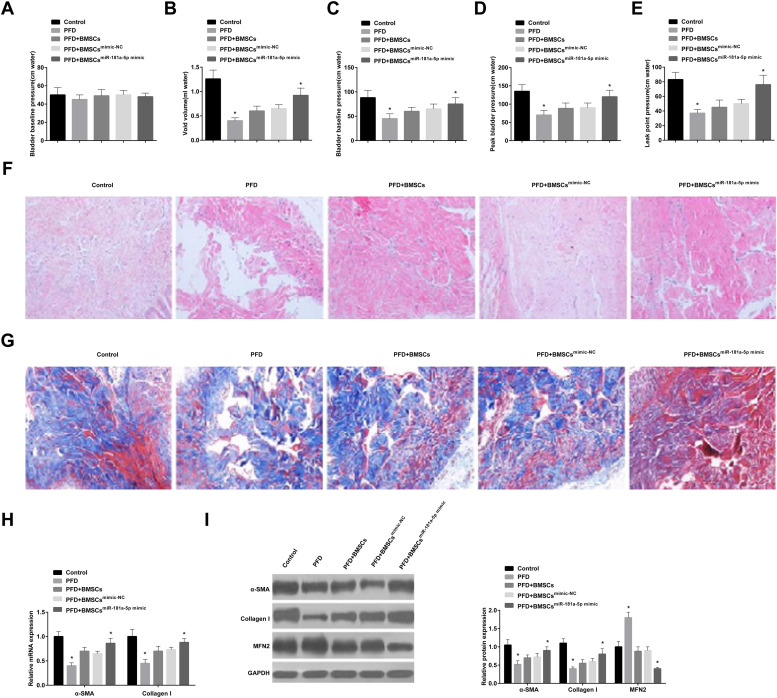
Animal model experiment. (A) Bladder baseline pressure in rats; (B) Void volume; (C) Bladder baseline pressure; (D) Peak bladder pressure; (E) LPP levels; (F) HE-staining results; (G) Masson staining results; (H/I) Evaluation of α-SMA and Collagen I in rats; * *p <* 0.05, *n =* 6.

The effect of BMSCs delivery of miR-181a-5p-mimic on PFD symptoms was explored. Baseline bladder pressure was found to be similar in all rats before the experiment (Fig. 5 A), but PFD rats had lower urinary output and peak bladder pressure (Fig. 5 B, C). In addition, PFD rats had lower peak bladder pressure and LPP (Fig. 5 D, E). Overall, PFD was successfully modeled in rats.

BMSCs or mimic-NC-modified BMSCs slightly elevated void volume and peak bladder pressure and had little effect on LPP in PFD rats, and miR-181a-5p mimic-modified BMSCs reduced void volume and enhanced peak bladder pressure (Fig. 5 B, C) and increased peak bladder pressure and LPP (Fig. 5 D, E). HE staining results showed that the muscle fibers of the urethra and surrounding tissues of control rats were densely arranged, and the muscle layer was intact and pink; while the muscle layer of the urethra wall of PFD rats was damaged, thinned, disordered, loose, and atrophy; transplantation of BMSCs improved the arrangement and density of muscle fibers and tightened the fascia muscle layer and connective tissue; in addition, the structure of muscle fibers was completely restored after treatment with miR-181a-5p mimic-loaded BMSCs (Fig. 5 F). Masson staining displayed that the collagen fibers were blue with uniform staining and larger staining area in control rats; the collagen fibers in PFD rats were light-blue, loose, and disordered, the proportion of connective tissue increased, and some blood vessel walls were thickened due to transparency; after BMSC transplantation, collagen staining was enhanced and arranged neatly; miR-181a-5p mimic transfection in BMSCs enhanced collagen expression and accelerated structural repair (Fig. 5 G). Finally, immunoblot analysis showed that MFN1 protein expression level was increased in PFD rats, while α-SMA and Collagen I levels indicated a significant decrease trend; injection of BMSCs or mimic-NC-treated BMSCs slightly reduced MFN1 protein expression, while the effect of miR-181a-5p mimic-loaded BMSCs was better; rats injected with BMSCs transfected with miR-181a-5p mimic had enhanced fibroblast differentiation of BMSCs (Fig. 5 H, I).

## Discussion

Since MSCs have shown great potential in soft tissue reconstruction,^23^^,^^24^ studies have focused on the role of BMSC therapy in PFD pathophysiology.^10^^,^^25^ In addition, certain miRNAs can positively regulate BMSCs to promote tissue repair.^26^^,^^27^ Therefore, the purpose of this study was to explore the effect of miR-181a-5p-modified BMSCs on PFD.

BMSCs had proliferation potential and possessed adipogenic and osteogenic differentiation abilities. Consistent with these results, former studies have also elucidated these functions of BMSCs.^28^^,^^29^ Fibroblasts can migrate to the wound area and proliferate, participate in wound contraction, extracellular matrix deposition, and tissue remodeling, and are essential for wound healing.^30^^,^^31^ Furthermore, previous studies have demonstrated that BMSCs can be internalized to regulate fibroblast differentiation.^32^^,^^33^ This work found that BMSCs differentiated into fibroblasts under certain induction conditions and BMSC transplantation improved LPP and CMG outcomes in a rat model of PFD, which was also observed in a previous study.^34^ Currently, the potential of BMSCs in soft tissue repair and reconstruction has been a hot topic, and BMSCs in damaged tissue can repair fascia in tissue-forming cells.^35^^,^^36^ As expected, BMSCs further increased collagen expression, repaired tissue structure, and tightened muscle and fascia connective tissue, demonstrating that BMSC transplantation is effective in the treatment of PFD. miR-181a-5p is ubiquitous in different tissues and cells and regulates various pathophysiological processes by targeting mRNA. miR-181a-5p has rich expression in mouse BMSCs.^37^ The present work indicated that fibroblast induction elevated miR-181a-5p expression. miR-181a-5p overexpression promoted fibroblast differentiation in BMSCs and enhanced fibroblast-related gene expression. *In vivo* experiments further elucidated that miR-181a-5p combined with BMSCs could further improve PFD in rats. Fibrous scaffolds cooperated with connective tissue can improve fibroblast differentiation of BMSCs,^38^ and miR-181a-5p tightened connective tissue, which further suggests that miR-181a-5p can promote the fibroblast differentiation of BMSCs. miR-181a-5p could regulate MFN1 gene expression in BMSCs at the post-transcriptional level, and MFN1 protein expression was down-regulated after BMSCs fibroblast induction. MFN1, a mitochondrial outer membrane protein, mediates mitochondrial fusion^39^ and is involved in the process in which miR-181c attenuates oxidative stress-mediated BMSC injury.^40^ However, to the authors’ knowledge, whether MFN1 mediates the process by which miR-181a-5p promotes fibroblast differentiation of BMSCs remains unclear. To this end, the study showed that MFN1 knockdown promoted the fibroblast differentiation of BMSCs, and abolished the effect of miR-181a-5p inhibition on the fibroblast differentiation of BMSCs. In addition, animal experiments reported that PFD rats injected with miR-181a-5p-overexpressed BMSCs exhibited higher MFN1 protein expression, suggesting that miR-181a-5p may act by targeting MFN1 expression.

Through this study, a novel regulatory mechanism for PFD therapy was identified. Nonetheless, this study is only a preliminary experiment of miR-181a-5p and MFN1 in fibroblast differentiation of BMSCs during PFD, and further, *in vivo* rescue experimental studies are needed to confirm the present findings. Also, the specific mechanism of PFD treatment is still unclear, and further empirical studies are still needed.

## Conclusion

The present study found that BMSCs containing miR-181a-5p regulate MFN1 expression during PFD recovery. Up-regulation of miR-181a-5p and down-regulation of MFN1 promoted fibroblast differentiation of BMSCs. miR-181a-5p combined with BMSC injection further enhanced the fibroblast differentiation of BMSCs and repaired tissue structure by down-regulating MFN1, highlighting miR-181a-5p as a new target for future PFD therapy.

## Ethics statement

The animal experiment research protocol was approved by the Ethics Committee of Muping District Hospital of Traditional Chinese Medicine (n° MP20190621) and performed in accordance with the “Guidelines for the care and use of experimental animals.”

## Authors’ contributions

Y.Z. conceived and designed the study. H.Y. analyzed the data. J.L. contributed to the literature review. Y.Z. wrote the manuscript. J.L. reviewed and edited the manuscript. All authors read and approved the final manuscript.

## Conflicts of interest

The authors declare no conflicts of interest.

## Data Availability

The figures and tables used to support the findings of this study are included in the article.
